# Women’s preferences for antenatal care in Tanzania: a discrete choice experiment

**DOI:** 10.1186/s12884-022-04634-x

**Published:** 2022-04-07

**Authors:** Elizabeth M. Camacho, Rebecca Smyth, Valentina Actis Danna, Deborah Kimaro, Flora Kuzenza, Rose Laisser, Paschal Mdoe, Livuka Nsemwa, Happiness Shayo, Tina Lavender

**Affiliations:** 1 School of Health Sciences, University of Manchester, Manchester, UK; 2 Centre for Childbirth, Women’s and Newborn Health, Department of International Public Health, Liverpool School of Tropical Medicine, Liverpool, UK; 3 Catholic University of Health and Allied Sciences, Mwanza, Tanzania; 4 Department of Research, Haydom Lutheran Hospital, Mbulu, Tanzania

**Keywords:** Antenatal care, Discrete choice experiment, Maternity services

## Abstract

**Background:**

The stillbirth rate in Tanzania remains high. Greater engagement with antenatal care may help to reduce stillbirths. We investigated which characteristics of antenatal care clinics are preferred by pregnant women in Tanzania.

**Methods:**

We conducted an unlabelled discrete choice experiment (DCE) with think-aloud interviews. Participants were pregnant women, regardless of parity/gestation, from the Mwanza and Manyara regions of Tanzania. We asked participants to choose which of two hypothetical antenatal clinics they would rather attend. Clinics were described in terms of transport mode, cleanliness, comfort, visit content, and staff attitude. Each participant made 12 choices during the experiment, and a purposively selected sub-set simultaneously verbalised the rationale for their choices. We analysed DCE responses using a multinomial logit model adjusted for study region, and think-aloud data using the Framework approach.

**Results:**

We recruited 251 participants split evenly between the 2 geographical regions. Staff attitude was the most important attribute in clinic choice and dominated the think-aloud narratives. Other significant attributes were mode of transport (walking was preferred) and content of clinic visit (preference was stronger with each additional element of care provided). Cleanliness of the clinic was not a significant attribute overall and the think-aloud exercise identified a willingness to trade-off cleanliness and comfort for respectful care.

**Conclusion:**

Women would prefer to attend a clinic with kind staff which they can access easily. This study suggests that exploration of barriers to providing respectful care, and enabling staff to deliver it, are important areas for future investment. The DCE shows us what average preferences are; antenatal care that is aligned with identified preferences should increase uptake and engagement versus care which does not acknowledge them.

**Supplementary Information:**

The online version contains supplementary material available at 10.1186/s12884-022-04634-x.

## Background

Every year around 2 million babies are stillborn (born with no signs of life, ≥ 28 weeks of pregnancy), and a further 2.4 million die in the neonatal period (death up to 28 days after birth) [1, 2]. However the burden of these deaths highlights stark inequalities, with an estimated 98% of perinatal deaths occurring in low- and middle-income countries (LMICs), and the greatest burden (over 75%), in sub-Saharan Africa and South Asia [1, 2]. Towards a global effort to tackle this, the ‘Every Newborn’ collaboration launched new targets for 2025, to end preventable stillbirths and newborn deaths [3]. A key target is that every woman should have eight antenatal contacts.

In Tanzania, most women attend at least one antenatal care visit (98%); only half (51%) attend four or more and there is an unacceptably high stillbirth rate (22.4 per 1000 births) [4]. The majority of stillbirths occur in younger first-time mothers (30%), women living in rural areas (78%), and women in the lowest wealth quintile (40%) [4]. Tanzanian women experience significant challenges attending antenatal care including lack of partner support, unwelcoming clinic environments, disrespectful care, perceived poor quality of care, socio-cultural beliefs, fear of HIV testing, poverty and distance from health clinics [5, 6]. Our long-term aim is to design antenatal services which better reflect the needs of pregnant women in Tanzania, with the hope that this increases engagement and reduces the number of stillbirths. But first, we need to know more about what women in Tanzania want their antenatal clinics to be like.

In an ideal world maternity services would be designed to meet everyone’s needs, but the reality of scarce human and financial resources means that compromises must be made. Discrete choice experiments (DCEs) can be used to better understand and quantify what is most important to the people using a particular service. This knowledge can help decision-makers to design a service which better meets the needs of service users, and in doing so has the potential to increase engagement with that service. In a DCE, participants are asked to make a choice between two different imperfect healthcare scenarios. The choice they make indicates how willing they are to trade-off between different attributes of the scenarios, thereby indicating what their preferences are.

The majority of existing literature on DCEs in relation to maternity care in LMICs has focussed on preferences for intrapartum care, generally with the aim of understanding how to encourage more women to birth in healthcare facilities [7–12]. Only one prior DCE regarding antenatal care was identified. It was conducted with women in Bangladesh and reported that consistent access to a female doctor, availability of branded drugs, respectful provider attitudes, and availability of Caesarean section delivery were important to respondents [13]. In this paper we report a DCE designed to identify preferences for antenatal care among pregnant women in Tanzania.

## Methods

### Setting participants and recruitment

This DCE was part of a larger research programme aiming to improve maternity care and better understand how to reduce the stillbirth rate in six countries in sub-Saharan Africa (NIHR Global Health Research Group on Stillbirth Prevention and Management in Sub-Saharan Africa; https://www.lstmed.ac.uk/research/departments/international-public-health/stillbirth-prevention-in-sub-saharan-africa-0). The DCE was conducted with pregnant women living in the catchment area for two maternity centres in Tanzania – Mwanza (largely urban) and Manyara (rural). The inclusion criteria were deliberately broad and included pregnant women aged over 18 years who had the capacity to give informed written consent.

Participants were approached when they attended the maternity centres for routine antenatal care, and the DCE was conducted at the centres. In order to also include the perspectives of unbooked women (i.e. those who did not attend the clinics), community health workers identified and introduced them to our team; these participants completed the DCE in their own homes. All participants were provided with a study information sheet and gave their consent before beginning the DCE. The demographic characteristics of participants was monitored during recruitment to enhance the representative of the sample. Partway through recruitment it was identified that younger mothers were underrepresented in the sample and so they were purposively targeted thereafter.

### Selection of attributes and levels

The attributes and levels included in the DCE are summarised in Table 1. Based on qualitative interviews conducted as part of the broader research programme a long list of potential attributes for the DCE was generated [14, 15]. The attributes on this list were: cleanliness of clinic, ease of travel (distance, availability of transport), facility comfort, waiting time to be seen, incentive to attend (e.g. food for mother, clothes for baby), attitude of staff, and content of clinic visit. In order to avoid overburdening participants only 5 attributes were to be included in the DCE, therefore it was necessary to identify the five attributes most likely to influence clinic preference.

**Table 1:** Attributes and levels included in the DCE

Attribute	Levels
Transport	Walk
	Bicycle/motorbike
	Public transport (bus)
	Private car/taxi
Cleanliness	Not clean enough
	Clean enough
Comfort	Seats
	Shaded waiting area
	Drinking water
	Toilet/washroom
Content	Conversation with a healthcare provider
	Conversation with a healthcare provider and physical examination
	Conversation with a healthcare provider, physical examination, and birth preparedness education (taught about giving birth)
Staff attitude	Harsh and rude
	Kind and friendly (respectful care)

Two community engagement and involvement groups (i.e. members of the public with lived experience of using maternity services; equivalent to ‘patient and public involvement’ groups) were asked to discuss and reach a consensus on which were the most important attributes to their group. Both groups selected content of clinic visits and facility comfort, therefore these were included in the DCE. Both groups also indicated that both distance to the clinic and availability of transport were important but to avoid having two attributes which measured the same construct, a new attribute was created. Mode of transport combines both the distance and ease of journey, for example a clinic which can be walked to is closer than one which would require a bus journey. Whereas a clinic which was reachable by bus or other modes of public transport may be far away, it would still need to be somewhere reasonably close to an existing public transport route. Conversely, a clinic which was only reachable by private car or taxi would be far away, difficult to get to, and may be prohibitively expensive or inaccessible for some women. Neither of the community engagement and involvement groups selected waiting time to be seen, cleanliness, incentive to attend, or attitude of staff. Previous qualitative work as part of this broader programme of research had identified attitude of staff as particularly important and to a lesser extent, cleanliness. Published DCEs exploring preferences of Tanzanian women for care during childbirth itself also suggested that staff attitude was important [7, 10] therefore staff attitude and cleanliness were included in the DCE. There was nothing to suggest that waiting time or an incentive for attending the clinic were priority attributes therefore these were not included in the DCE.

We piloted the survey with women in Tanzania to ensure that the levels selected were meaningful and clear in the local context of the DCE. We used feedback from the pilot surveys to improve the clarity of how the levels were defined and described.

### Study design, sample size, and survey

As shown in Table 1, the experiment included two attributes with 2 levels, one with 3 levels, and two with 4 levels. This gives a total of 192 (i.e. 2^2^ × 3 × 2^4^) possible combinations of attributes. The *dcreate* command in STATA software (version 15; StataCorp LP, College Station, USA) was used to produce a D-efficient design of 24 choice sets, which was organised as 2 blocks of 12 sets (using the *blockdes* command) so that participants were only asked to complete 12 choice tasks, thereby minimising burden. According to the rule of thumb proposed by Johnson and Orme [16], based on the number of choice tasks (*n* = 12), number of alternatives (*n* = 2), and number of analysis cells (as this is a main effects design, this is equal to the largest number of levels for any attribute) (*n* = 4), a sample size of at least 83 would be sufficient for this DCE. The study aim was to recruit 1.5 times this number of participants to complete each block of choice tasks, to allow for any missing data or partially completed surveys.

The survey itself started with an introductory example, followed by 12 unlabelled choice sets. It took participants approximately 30–45 min to go through the instructions and complete the survey. Each attribute was described in written Swahili (translated from English by author PM) and a simple icon to enable participants of low literacy to engage with the survey (see Supplementary Material for example choice task). The pen-and-paper survey was conducted in Swahili by trained research midwives, who gave instructions to participants using a standardised script.

### Statistical analysis

A random sample of 20 records (10 per site) was double-entered to validate data. A margin of error of 0.6 (Manyara) and 0.3 (Mwanza) was detected. Errors were corrected and no further action was needed. Data were analysed using STATA software. Descriptive statistics were calculated for the sample and reported as mean (standard deviation), median (range), or percentage, as per the variable characteristics. A mixed logit model, which included study site as a fixed effect and all of the attributes as random effects (to allow for the random distribution of attribute coefficients), was used to analyse participant choices [17].

The results of the whole sample model are reported as β coefficients, with standard errors, *p*-values, 95% confidence intervals, and standard deviations. The *p*-values and 95% confidence intervals both indicate whether a particular attribute had a statistically significant impact on choice, however both are reported to aid comprehension. The coefficients indicate the mean relative preference for each attribute, conditional on the other attributes in the choice set [10]. The sign of the β coefficient indicates whether an attribute is preferred (positive) or unpreferred (negative). The β coefficient, standard deviation, and normal cumulative distribution function were used to calculate the proportion of the sample for whom each attribute has a positive effect on clinic choice [18].

Sub-groups, based on demographic characteristics, which were hypothesised a priori to influence preferences for antenatal care were also explored. These were: urban/rural dwelling, level of education, and gravidity.

### Think aloud interviews

To gain greater insight into participants’ reasons for choice preferences, the think-aloud approach was used [19]. A sub-set of women were purposively selected from the larger sample to ensure maximum variation. The sample included women from a range of ages, parities, and geographical locations (urban and rural); we also targeted women who were booked and unbooked for health facility care. Sample size was guided by data saturation, i.e. no new findings were emerging. Participants were encouraged to verbalise their thoughts whilst simultaneously responding to the choice tasks. Prompts were used throughout the process, by trained researchers. The think-aloud interviews were audio recorded and field-notes were kept.

Verbatim transcripts were analysed by four authors (TL, VAD, RL, RS), using a charting approach [20], with the DCE attributes as the guiding framework. Individual responses were mapped onto the chart, alongside the quotational evidence which supported the decisions made. During the process, we looked for any changes in preferences to determine whether views were influenced by the experiment. We also explored whether participants considered all attributes or whether their a priori views resulted in a strong desire for a particular attribute.

## Results

Of the 254 eligible women approached, 251 (99%) consented to complete the DCE survey. The demographic characteristics of the 251 participants are reported in Table 2. The mean age of the sample was 27 years, and just over a quarter of participants were primiparous. The study sites were selected in order to include participants from both rural and urban areas, therefore it was expected that there would be some differences between the participants. At Mwanza, around 20% of the participants lived in rural areas compared with 100% of those at Manyara. In Mwanza, more than half of the participants had completed education to or beyond secondary school level and almost 20% were formally employed, versus 15% and 5% respectively in Manyara. Seven participants across the two sites had no contact with antenatal care in this pregnancy (i.e. were unbooked). There were no participants who chose the same clinic for each choice set, suggesting that participants had sufficient understanding of the task to avoid this type of constant preference [21].

**Table 2:** Demographic characteristics of survey participants

	**Whole sample** (*n* = 251)	**Mwanza** (*n* = 126)	**Manyara** (*n* = 125)
**Age (years)**			
Mean (SD)	27.1 (7.4)	28.0 (8.1)	26.2 (6.5)
Median, min–max	25 (18–47)	27 (18–43)	24 (18–47)
**Province of residence**			
Urban	101 (40.2%)	101 (80.2%)	-
Rural	150 (59.8%)	25 (19.8%)	125 (100%)
**Civil status**			
Married / living with partner	214 (85.3%)	100 (79.4%)	114 (91.2%)
Single	35 (13.9%)	24 (19.0%)	11 (8.8%)
Divorced/Widowed	2 (0.8%)	2 (1.6%)	-
**Education**			
Never been to school	18 (7.2%)	6 (4.8%)	12 (9.6%)
Primary	148 (58.9%)	54 (42.9%)	94 (75.2%)
Secondary or higher	85 (33.9%)	66 (52.3%)	19 (15.2%)
**Formally employed**			
Yes	30 (12.0%)	24 (19.0%)	6 (4.8%)
No	221 (88.0%)	102 (81.0%)	119 (95.2%)
**Previous pregnancies** (including miscarriages and stillbirth)			
Yes	183 (72.9%)	82 (65.1%)	101 (80.8%)
No (nulligravida)	68 (27.1%)	44 (34.9%)	24 (19.2%)
**Gravida***(women with previous pregnancy)*			
Median (min–max)	3 (1–10)	3 (1–9)	2 (1–10)
1–2 pregnancies	88	33 (40.2%)	55 (54.4%)
3–4 pregnancies	47	24 (29.3%)	23 (22.8%)
≥ 5 pregnancies	48	25 (30.5%)	23 (22.8%)
**Gestation at completion of questionnaire (weeks)**			
Median (min – max)	28 (12–38)	30 (18–37)	24 (12–38)

The findings from the DCE for the whole sample are summarised in Table 3. The attribute with the greatest magnitude of association with clinic preference was whether staff were kind and friendly (rather than harsh and rude) (β = 2.47, *p* < 0.001). There was also a clear preference for all other modes of transport when compared to a private car or taxi. Women demonstrated greater preference for clinic visits with greater content. Of the three levels of care in the choice sets (1 – conversation with healthcare professional, 2 – conversation plus physical examinations, 3 – conversation, examination, plus education in birth preparedness), compared to having a conversation with a healthcare professional alone, there was a significant preference for also having a physical examination (β = 0.20, *p* = 0.021) and for having an examination and birth preparedness education (β = 0.68, *p* < 0.001), with the magnitude of the preference for the highest level of care being the highest. Whether or not the clinic was clean did not have a significant impact on clinic choice. Preferences were less clear in terms of the comfort of the clinic. When compared with availability of seating (the least preferred level), the magnitude of the association with clinic choice was similar for availability of toilet facilities, shade, and drinking water. The coefficient for drinking water was marginally higher than the others and the preference for toilet facilities and shade both bordered statistical significance (*p* = 0.055 and *p* = 0.048 respectively).

**Table 3:** Mixed logit model results for whole sample

Attribute	beta	SE	*p*-value	95% CI	SD	% Pos
Constant	-0.039	0.127	0.758	-0.287–0.209		
Site	-0.231	0.119	0.053	-0.464–0.003		
Transport (versus car/taxi)						
Walk	1.628	0.139	< 0.001	1.355–1.901	0.487	99.96%
Bike	1.164	0.122	< 0.001	0.924–1.403	0.008	100.00%
Public transport	1.004	0.131	< 0.001	0.748–1.261	0.189	100.00%
Cleanliness (versus not clean enough)						
Clean enough	0.086	0.072	0.23	-0.055–0.228	0.084	84.70%
Comfort (versus availability of seating)						
Toilet facilities	0.247	0.129	0.055	-0.005–0.500	0.221	86.81%
Shade	0.26	0.131	0.048	0.003–0.517	0.347	77.32%
Drinking water	0.307	0.135	0.022	0.448–0.569	0.405	77.58%
Content of clinic visit (versus talking to healthcare professional only)						
Talking and physical examination	0.2	0.087	0.021	0.030–0.371	0.009	100.00%
Talking, examination, and education	0.683	0.103	< 0.001	0.481–0.885	0.371	96.72%
Staff attitude (versus harsh and rude)						
Kind and friendly	2.472	0.134	< 0.001	2.209–2.736	0.988	99.38%
Model information						
Number of observations	3012					
Log likelihood	-1040					
Likelihood ratio statistic	89.9					

% Pos   Proportion of the sample for whom the attribute had a positive effect on their choice

As they were considered to be improvements (i.e. versus the respective comparator levels) it was expected that the attributes in the model would have a positive effect on choice (i.e. make people more likely to choose a clinic with that attribute) for the majority of participants. The attributes related to cleanliness and comfort had a positive effect on choice for the smallest proportion of the sample.

The DCE results for different sub-groups of the sample are summarised in Table 4. Although there is not a formal sample size calculation for DCEs as the sub-groups contain fewer participants than the overall sample, the results (in particular the statistical significance) from these analyses should be interpreted with caution. In all sub-groups, the attribute with the greatest magnitude of preference was kind and helpful staff. Participants from urban areas were very consistent in choosing the clinic with kind and helpful staff regardless of other attributes (i.e. in 96% of choices). Women from urban areas showed a preference for clean clinics and availability of a shaded waiting area, neither of which were significant attributes for the sub-group of women from rural areas. There were no major differences between the preferences of women in the different education level sub-groups, although the size of the preference from kind and friendly staff was notably larger in women who had completed higher levels of education. There were more differences between the group of women for whom this was their first pregnancy and those with previous pregnancies. Women who had not been pregnant before showed a preference for the clinic to be clean and for availability of seating (compared to drinking water i.e. there was a negative beta coefficient for drinking water).

**Table 4:** Mixed logit model results for key sub-groups

	**Urban**		**Rural**		**Up to Primary**		**Secondary or higher**		**First pregnancy**		**Not first pregnancy**	
**Attribute**	**beta**	**95% CI**	**beta**	**95% CI**	**beta**	**95% CI**	**beta**	**95% CI**	**beta**	**95% CI**	**beta**	**95% CI**
Constant	7.162	-0.15–14.47	0.099	-0.21–0.41	-0.039	-0.34–0.26	0.491	-0.30–1.29	0.259	-0.53–1.05	0.057	-0.24–0.36
Site		n/a	-0.293	-0.80–0.21	-0.11	-0.43–0.21	-0.311	-1.10–0.48	0.217	-0.67–1.10	-0.186	-0.49–0.12
Transport (versus car/taxi)												
Walk	15.287	2.27–28.30^a^	2.694	2.19–3.20^a^	2.207	1.80–2.61^a^	1.222	0.38–2.07^a^*	1.464	0.58–2.35^a^	2.056	1.67–2.45^a^
Bike	18.844	3.45–34.24^a^	1.727	1.34–2.12^a^	1.518	1.19–1.84^a^	1.189	0.40–1.98^a^	1.764	0.91–2.62^a^	1.368	1.05–1.69^a^
Public transport	11.345	1.31–21.38^a^	1.539	1.17–1.91^a^	1.34	1.00–1.68^a^	0.766	0.07–1.47^a^	0.774	-0.10–1.65	1.302	0.97–1.63^a^
Cleanliness (versus not clean enough)												
Clean enough	5.127	0.55–9.71^a^	0.100	-0.12–0.32	0.03	-0.15–0.21	0.299	-0.22–0.82	0.713	0.09–1.34^a^	0.041	-0.14–0.22
Comfort (versus availability of seating)												
Toilet facilities	-0.392	-4.41–3.62	0.282	-0.05–0.62	0.305	-0.02–0.63	0.505	-0.17–1.18	0.095	-0.72–0.91	0.318	0.01–0.63^a^
Shade	9.891	0.66–19.13^a^	0.221	-0.15–0.59	0.185	-0.15–0.52	1.144	0.30–1.99^a^	0.381	-0.48–1.25	0.31	-0.02–0.64
Drinking water	-0.225	-3.99–3.54	0.345	-0.01–0.70	0.311	-0.02–0.64	0.402	-0.41–1.22	-0.187	-1.00–0.62	0.438	0.11–0.76^a^
Content of clinic visit (versus talking to healthcare professional only)												
Talking and physical examination	6.279	-0.08–12.63	0.343	0.10–0.58^a^	0.235	0.02–0.45^a^	0.522	-0.07–1.11	0.667	-0.05–1.39	0.213	0.01–0.42^a^
Talking, examination, and education	9.829	1.25–18.40^a^	1.139	0.77–1.51^a^	0.832	0.55–1.11^a^	1.121	0.35–1.89^a^	1.197	0.27–2.12^a^	0.841	0.56–1.13^a^
Staff attitude (versus harsh and rude)												
Kind and friendly	56.983	10.36–103.6^a^	2.430	1.95–2.91^a^	2.494	2.08–2.91^a^	5.787	3.96–7.62^a^	5.744	3.35–8.14^a^	2.749	2.33–3.12^a^
Model information												
Number of observations	2424		3600		3984		2040		1632		4392	
Log likelihood	-128		-666		-711		-210		-190		-745	
LR statistic	78.3		236.4		165.5		78.0		89.9		174.2	

^a^statistically significant (95% CI does not include 0); all participants from Urban areas were recruited from the same study site. *LR*  Likelihood ratio

Significant attributes in the whole sample were: walking, bike, public transport, shade, drinking water, physical examination, birth preparedness education, kind staff

### Think-aloud

Twenty-eight participants took part in think-aloud interviews; 19 resided in a rural and 9 resided in an urban location. All seven of the unbooked participants took part in think-aloud interviews. Ages ranged from 19 to 43 years. The majority were multigravida (*n* = 25, 89%). Most participants (*n* = 24) considered and commented on all attributes, with the remainder making trade-offs between at least two attributes. Examples of participants’ trade-off reflections and factors influencing choice of clinic are reported in Supplementary Material (Table S1).

The narratives were dominated by the need for respectful care and easily accessible transport; however, participants were prepared to trade their ability to walk or use public transport, for ‘friendly nurses’ who ‘were polite and keep secrets’ (Table S1). Women were prepared to find the funds for private journeys if they would be guaranteed a ‘good service.’ Women interviewed would also trade-off cleanliness and comfort for respectful care. Women’s preferences were heavily influenced by their own or others’ experiences and a desire for ‘value for money’. Women who were unbooked were influenced by their belief in traditional medicine, their fear of being discriminated against (due to single status, being an older woman) and the misconception that there are ‘less diseases in the village’.

## Discussion

Pregnant women in two sites in Tanzania were involved in a DCE study, where they were asked to choose which of two hypothetical antenatal clinics they would rather attend. The clinics were different in their attributes of: mode of transportation to get to the clinic, cleanliness, comfort, content of the clinic visit, and staff attitude. The most important attribute in determining choice of antenatal clinic was staff attitude. Women across all sub-groups showed a preference for staff who were kind and friendly compared with those who were harsh and rude; this was also reflected in the think-aloud results. Both the DCE and think-aloud also showed that a clinic which women could easily access (e.g. walking distance) was preferable. Cleanliness of the clinic was not a significant preference for the sample as a whole and the think-aloud confirmed that women would rather trade-off cleanliness for kinder staff. However, first-time mothers and mothers from an urban area did show a preference for a clean clinic. Across all sub-groups women showed the greatest preference for clinics where they would receive the most care (talking to a healthcare professional, a physical examination, and birth preparedness education).

### Strengths and limitations

There are a number of strengths to this analysis, the DCE was designed according to published guidance for general DCE design and analysis and specific guidance for doing so in low- and middle-income countries [18, 22, 23]. The results of the think-aloud exercise support the findings from the DCE and demonstrate that participants made trade-offs between attributes to inform their choices. Attributes and levels were selected and defined through published qualitative literature [5, 6], and with input from community engagement and involvement groups. The choice tasks were translated into Swahili and represented using simple pictures so that participants with lower levels of literacy could still participate. Although this study was only conducted in one country, it included participants from 2 geographical areas (one rural and the other predominantly urban), and the findings were similar to other related studies from the region [7, 10]. There are even similarities in the results with a DCE conducted in the UK which indicates that there may be some preferences for antenatal care which are important across a broad range of settings [24].

Another strength was that all respondents answered all questions on the DCE. This suggests that the number of choice sets (*n* = 12) did not overburden participants and that it was an acceptable task (i.e. participants were not so unduly distressed by the DCE that they could not complete it). However it was necessary to make a number of trade-offs in order to ensure that the DCE was not too long or too complex. As described in the methods section, there was a longer list of potentially important attributes which could have been included in the DCE. Although we sought input from service users in refining the final list of attributes, it is possible that some key things which would influence behaviour in a real-world setting are not included in the DCE. To avoid overcomplicating the DCE we did not include an ‘opt out’ option whereby participants can choose neither of the hypothetical clinics. This may have introduced some bias as participants were “forced” to choose a clinic which in reality they may not attend. The results of the DCE do not allow us to infer actual behaviour [25]; would kind and respectful staff actually result in more women engaging with antenatal care? In addition to this, and as noted by other studies conducted with women in Tanzania [5, 10, 26], pregnant women themselves may not be the decision maker in their antenatal care. It does however provide some guidance for trying to better match the provision of antenatal care to the preferences of pregnant women which can be used to enrich evidence from other studies [27].

DCEs can be a useful tool to generate quantitative evidence to support decision-makers trying to ensure that healthcare services meet the requirements of the people who use the service. However, there are other preference-elicitation approaches which could potentially have been used, for example time trade off or standard gamble surveys. However, both approaches require participants to be able to understand and interpret more complex instructions and concepts (e.g. probability) than required for DCEs. We opted for a DCE design in this setting as we did not want to exclude women with a low level of literacy. A limitation of DCEs in general is that the results may be sensitive to the specific language used to describe the levels within an attribute. For example, our finding that staff attitude was by far the most important attribute to participants, may also reflect that this attribute was described in terms of two almost diametrically opposed levels, whereas the distinctions between the levels of other attributes were more subtle. Herein participants’ ‘decisions’ may have been somewhat guided by the attribute descriptions used. A specific limitation related to the selection of the attribute levels, was for clinic comfort. The levels (drinking water, shade, seating, toilet facilities) were unrelated items rather than representing increasing levels of comfort. This enabled us to maintain the simplicity of the DCE, however no clear choice was identified in terms of which element of comfort is most important in determining clinic choice. This may reflect that pregnant women in Tanzania do not have a clear preference for any of the levels offered, or that they value other attributes more highly than comfort. Another way we tried to maintain simplicity in the DCE was by not including an attribute with continuous (i.e. numeric) levels. However, this meant that it was not possible to estimate trade-offs between attributes. Another limitation of the DCE was that a specific quality control option was not included in the choice set, which makes it difficult to assess the robustness of the data collected. In terms of the analysis, a mixed logit model was used to analyse the preference data. The benefit of this approach is that it allows for unobserved heterogeneity in responses (i.e. unidentified sub-groups who may respond differently) but the downside is that is does not allow the identification of where preferences differ [17].

Although efforts were made to capture the preferences of women who had not attended the antenatal clinic at all, we were only able to recruit 7 participants from this group. The difficulty in recruiting more participants from this group reflects the high proportion of women in Tanzania who attend at least one antenatal care visit (98%) [4]. However, as only half of women attend four or more times [4] (with presumably fewer still meeting the ‘Every Newborn’ goal of 8 visits), our sample of women with some contact is representative of the population whose preferences we aimed to elicit.

### Interpretation

Respectful care is a fundamental human right and a key determinant of quality care during childbearing. Evidence shows that disrespect and abuse remain prevalent, primarily in LMICs [28]. The long-term impacts of disrespectful care on physical and psychological health outcomes can have a negative impact on the family and wider community, in terms of women’s inability to contribute at a societal level and through economic hardship. Two previous DCEs of the preferences of women in Tanzania in relation to intrapartum care (rather than antenatal care) reported similar findings to the present study [7, 10]. Both studies found that staff attitude/kindness was the most important predictor of hospital preference. One of the studies also reported, similarly to our findings, that kindness was a greater predictor of preference than cleanliness [10]. Although these studies relate to a different stage of pregnancy than the present study, and include some different attributes, a strikingly similar and clear message about the preferences of pregnant women in Tanzania is apparent.

Although in a very different setting and population, a DCE of antenatal care conducted with primiparous women in the UK NHS reported that women preferred antenatal visits which included additional birth preparedness education and an opportunity to ask questions about the pregnancy/baby compared with routine check-ups alone [24].

The UK study also reported that women preferred antenatal visits to take place in their local community. Although there was not a direct attribute for this in the present study, the clear preference for being able to get to the clinic on foot suggests that this another common preference. Whilst not the same population, a DCE of preferences for HIV testing clinics conducted with men and women in an urban setting in Tanzania, reported that distance to clinic was the most important attribute [25].

## Conclusions

There is a clear finding that receiving respectful care from kind and friendly staff is the most important attribute to pregnant women in Tanzania for their in-clinic antenatal care. Despite the adage that “manners cost nothing”, this study provides evidence for the potential benefit of trying to better understand barriers for staff in delivering respectful care and of investing in staff to enable them provide it. Women also preferred clinics which they could walk to. Especially in settings where people may live very far from centralised hospitals, the feasibility of setting up local or regional or mobile clinics should be explored. Although the DCE does not predict real-world behaviour, it is reasonable to think that by better aligning antenatal care with the preferences of pregnant women, they will be more likely to attend and engage with that care.

## Supplementary Information

**Additional file 1. **

## Data Availability

The datasets used and/or analysed during the current study available from the corresponding author on reasonable request.

## Abbreviations

DCE: Discrete choice experiment
LMIC: Low and middle-income country
NIHR: National Institute for Health Research
