# Supplementary material for: Women’s preferences for antenatal care in Tanzania: a discrete choice experiment
Source: BMC Pregnancy Childbirth. 2022 Apr 7;22:296. doi: 10.1186/s12884-022-04634-x (PMC8991681; doi:10.1186/s12884-022-04634-x)
Supplement: Supplementary file 1 — Additional file 1. [file 12884_2022_4634_MOESM1_ESM.docx]

**SUPPLEMENTARY MATERIAL**

**Women’s preferences for antenatal care in Tanzania: a discrete choice experiment**

Elizabeth M Camacho, Rebecca Smyth, Valentina Actis Danna, Deborah Kimaro, Flora Kuzenza, Rose Laisser, Paschal Mdoe, Livuka Nsemwa, Happiness Shayo, Tina Lavender

**Supplementary Figure 1.** Sample choice task from the DCE

| 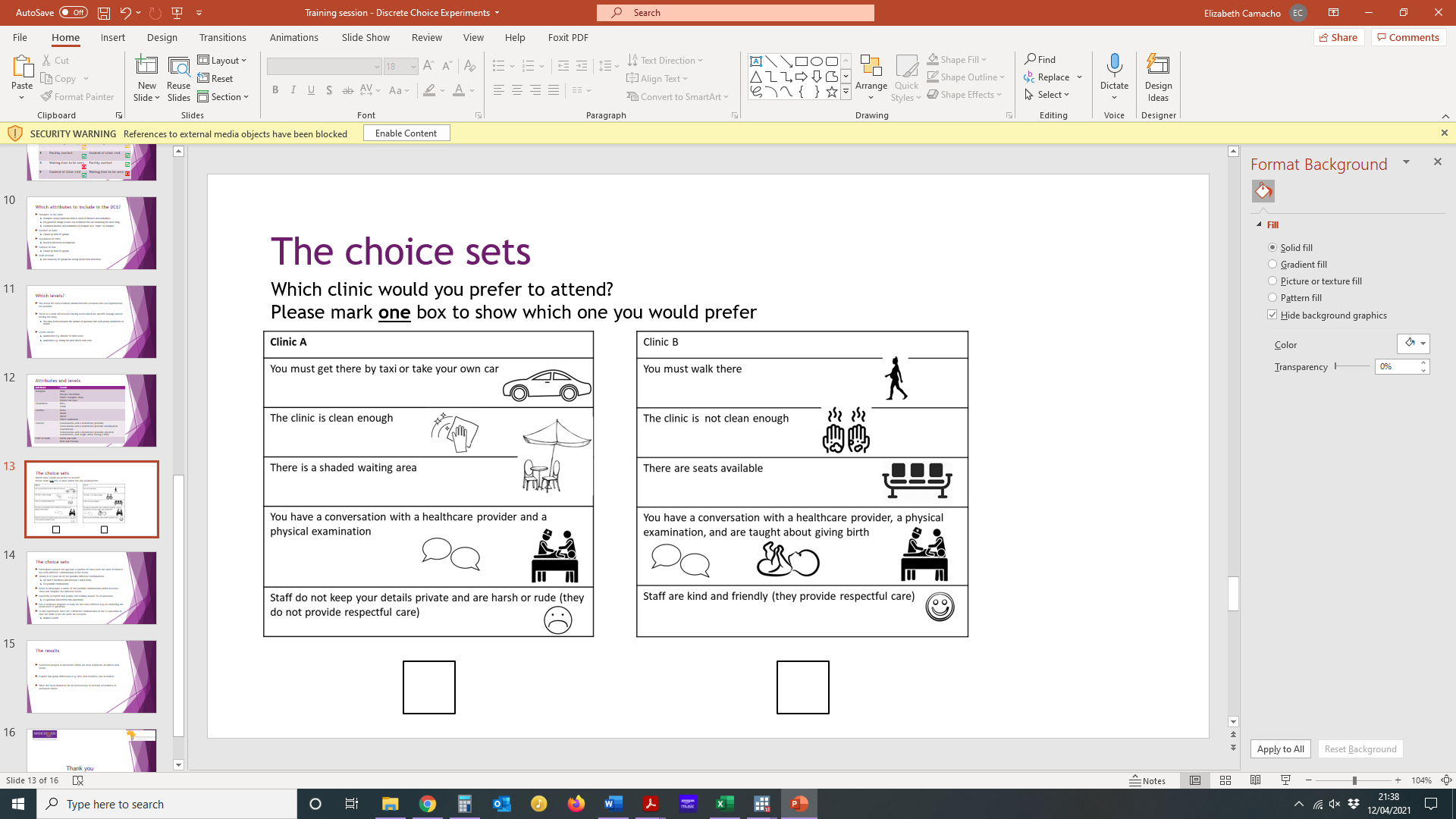 |
| --- |

**Table S1.** Think aloud responses

| **Attribute** | **Examples of women’s choice justifications: Verbatim quotes** |
| --- | --- |
| Staff attitude | “If you are getting good services at a clinic that is far, you will see the place is just near because you are getting what you want and what you like most, also you won’t feel that you are tired because the service you are receiving makes you feel at peace. If you are not treated well at the clinic, even if the clinic is near, you won’t see as it is near because you are not comfortable with the place. I won’t mind walking for a long distance but I go and receive good services, where the nurses are polite, they use good language. [Woman, Rural]  'I am ready to go to a far clinic so that I can be served with polite nurses’ [A-076, Rural] |
| Transport | ‘I chose this clinic because of my low-income; I can afford to pay for a bicycle. What most took me there are the services that they provide, the polite language used by health providers.’ [C-015, Urban]  ‘Some women don’t have the money to go to the clinic as they will need money for transport…But if the nurses use polite language, women will find the means of going to the clinic’ [C-015, Urban] |
| Content | ‘I will use money so that I can get good services. Get laboratory tests, education about child health care and maternal and health workers are polite…I will just use money to reach the hospital, which is okay too’ [B-017, Rural] |
| Cleanliness | ‘I will go to clinic B…I do agree that the clinic is not clean but I can walk to the clinic…’ [A-099, Rural]  ‘I won’t mind that it is not clean, this is because I am not going to sit forever at this clinic.’ [A-035, Rural] |
| Comfort | ‘What I want is services and not the shade’ [A-118, Rural]  ‘We mostly look at the way the people treat you and not the way the environment is good. A mean nurse won’t give you good explanations….I won’t mind spending money to get good services and a nice nurse….I wouldn’t mind about dirty toilets…..’ [B-030, Urban] |
| **Influencers**  **Prior Experience:** “The services that I received earlier influenced me to choose this clinic again because I was comfortable with their service” [A-021, Rural]  **Experience of others:** ‘Nurses don’t keep secrets. My friend has met nurses who have not kept your secrets and she was not happy’ [A-118, Rural]  **Beliefs:** ‘We believe in traditional medicine. We don’t believe in white people’s things …. I deliver alone, or maybe I call a friend to help me, and you know what, in this village there are no diseases’ [C-039, Urban, Unbooked]  **Desire for value for money:** ‘I must have money to pay for the bus fare. I can struggle to get money but still the place where I am going has a problem…’ [C-063, Urban]  **Fear of discrimination:** It is a must that you go with your husband to the clinic; that has not encouraged me to attend….I wasn’t able to buy anything [baby layette]. This really discouraged me… I didn’t want the nurses to get angry and scold me.’ [A116, Rural, Unbooked] | |
